# The Relation of Diet and Health: You Are What You Eat

**DOI:** 10.3390/ijerph19137774

**Published:** 2022-06-24

**Authors:** Ann-Kathrin Lederer, Roman Huber

**Affiliations:** 1 Center for Complementary Medicine, Department of Medicine II, Medical Center—University of Freiburg, Faculty of Medicine, University of Freiburg, 79106 Freiburg, Germany; roman.huber@uniklinik-freiburg.de; 2 Department of General, Visceral and Transplantation Surgery, University Medical Center, 55131 Mainz, Germany

The intake of food is more than just a necessary process for ensuring the functionality of the human body. Nutritional components become a part of us, interacting with our gut microbiota, immune system and metabolism. For decades, it has been known that the occurrence and the course of a variety of typical Western diseases such as cancer, stroke and myocardial infarction are affected by diet [1,2,3,4]. The common Western diet consists of highly processed foods and is rich of animal protein, trans-fatty acids and sugar [5,6]. Recent research shows that a diet rich in red meat and processed meat leads to a high risk of cancer, especially colorectal cancer [7,8]. Substantial evidence indicates that eating more vegetables and fruits and less meat is able to decrease overall cancer risk and improve health [4,9,10,11]. Plant-based diets, in particular, are reported to be beneficial for health [12,13]. Remarkably, the definition of a “plant-based diet” is sometimes inconsistent across publications, which must be considered when evaluating results of nutritional trials [12].

Diet does not only affect the risk of cancer occurrence; it appears that it might also play a pivotal role in the prognosis and outcomes of cancer patients [14,15,16,17,18]. The gut microbiota might be a key contributor to these observations as animal models and observational human cohort trials emphasize the impact of gut microbiota on the toxicity and efficacy of anticancer immunotherapy and chemotherapy [19,20]. CTLA-4-specific antibodies are able to control the progression of sarcoma in specific pathogen-free mice, but not in germ-free mice or those treated with broad-spectrum antibiotics [19]. The interplay between gut microbiota and the immune system constitutes a sensitive equilibrium controlling billions of gastrointestinal inhabitants. The existence of these commensals is essential for the function of the immune system, for digesting foods and processing nutrients in a healthy human being [21,22]. The composition of gut microbiota is affected by a variety of external factors such as diet. In general, research emphasizes a high intra-individual stability of human microbial communities, but they can rapidly be individually altered by external factors [23,24,25]. Faith et al. were able to show that 60% of bacterial strains, which were isolated from the stool of 37 healthy individuals, remained stable for up to 5 years [23]. Even in the case of antibiotic treatment, the gut microbiota of healthy individuals is able to recover rapidly [26]. Nevertheless, several publications indicate associations between the gut microbiota composition and occurrence and development of diseases, but the distinction of potentially beneficial and potentially harmful gut microbiota is challenging [27,28,29]. The results of human microbiome trials are mostly population-based, showing a great variance of microbiota results, implying that the gut microbiome is as individual as its owner [27,30]. In 2020, Shanahan et al. postulated that the microbiome “is a reflection, in part, of host genetics, but mainly environmental and lifestyle factors, including ethnicity, geographic location, and diet” [30].

The underlying mechanism, how food affects the human body, is complex and not only explained by the composition of gut microbiota. Recent research also emphasizes (chronic) inflammation, e.g., caused by the disturbed interplay of diet and gut microbiota, as a driver of diseases [31,32,33,34]. In 2022, Tan et al. postulated that “diet appears to be one of the most influential environmental factors regulating regulatory T cell biology” [35]. This axis might also be responsible for the development of inflammatory bowel diseases [36]. Nutritional components such as dietary fibers and also the gut microbiota affect the differentiation and the survival of intestinal regulatory T cells [37,38]. Different nutritional components are able to provoke variable immune-modulating effects [34,39]. For example, Christ et al. reported sugar and saturated fatty acids to be pro-inflammatory, whereas omega-3-fatty acids and polyphenols are able to decrease inflammation [34]. Even plant-based diets can be healthy or harmful depending on composition [12,40]. Different plant-based diets differed significantly regarding cardiovascular risk reduction [41]. Furthermore, it is assumable that the interplay of different food compounds might be responsible for the observed beneficial effects of diet [31,35].

Our own experiences and the results of further clinical trials showed that plant-based diets are able to relieve the symptoms of patients with autoimmune diseases [42,43,44,45]. In a randomized-controlled pilot trial conducted in 2017 comparing healthy, young, previously omnivore participants, who were assigned to either a vegan diet or a meat-rich diet for 4 weeks, we were able to show a significant difference of leukocytes, thrombocytes and monocytes between vegan participants and meat-rich participants [46,47]. Interestingly, we did not observe a difference in FoxP3, a potential master regulator for the development and function of regulatory T cells, between a vegan diet and meat-rich diet in our trial.

In 2018, Witkamp et al. underlined that the effect of nutritional components is not comparable to the effect of drugs [48]. Nutritional therapies are not a pill to relieve symptoms, but a holistic approach to help the whole human body recover. It is said that, more than 2000 years ago, Hippocrates postulated nutrition as a remedy. Changing diet appears to be a simple and inexpensive way to improve a patient’s life. There is evidence for nutritional therapies to treat a broad spectrum of diseases, not only for Western diseases such as diabetes and hypertension, but also for critical ill patients and psychiatric patients [49,50]. In 2017, Kahleova et al. postulated that plant-based diets could be able to reduce the risk of coronary heart disease events by 40% and the risk of cerebral vascular disease events by almost 30% [51]. Therefore, it is justified to discuss whether it is ethically reasonable to neglect nutrition in medical training and everyday medical life [40]. Although the research of the recent decades has accentuated the overwhelming role of diet in health and disease, recent publications indicate many physicians’ just basic knowledge of diet [52,53]. In the future, we have to pay more attention to the diets of patients as nutrition appears to be a key contributor to health, longevity and delayed ageing [3,54,55].
